# A systematic review and network meta‐analysis of immunotherapy and targeted therapy for advanced melanoma

**DOI:** 10.1002/cam4.1001

**Published:** 2017-05-01

**Authors:** Joao Paulo da Silveira Nogueira Lima, Mina Georgieva, Benjamin Haaland, Gilberto de Lima Lopes

**Affiliations:** ^1^ Drug Development UnitInstitute of Cancer ResearchSuttonUnited Kingdom; ^2^ Stewart School of Industrial & Systems EngineeringGeorgia Institute of TechnologyAtlantaGeorgia; ^3^ Sylvester Comprehensive Cancer CenterGlobal Oncology ProgramMiamiFlorida; ^4^ Present address: Medical Oncology DepartmentAC Camargo Cancer CenterSao PauloBrazil

**Keywords:** Bayesian, biomarker, BRAF, immunotherapy, melanoma, meta‐analysis

## Abstract

Immune and BRAF‐targeted therapies have changed the therapeutic scenario of advanced melanoma, turning the clinical decision‐making a challenging task. This Bayesian network meta‐analysis assesses the role of immunotherapies and targeted therapies for advanced melanoma. We retrieved randomized controlled trials testing immune, BRAF‐ or MEK‐targeted therapies for advanced melanoma from electronic databases. A Bayesian network model compared therapies using hazard ratio (HR) for overall survival (OS), progression‐free survival (PFS), and odds ratio (OR) for response rate (RR), along with 95% credible intervals (95% CrI), and probabilities of drugs outperforming others. We assessed the impact of PD‐L1 expression on immunotherapy efficacy. Sixteen studies evaluating eight therapies in 6849 patients were analyzed. For OS, BRAF‐MEK combination and PD‐1 single agent ranked similarly and outperformed all other treatments. For PFS, BRAF‐MEK combination surpassed all other options, including CTLA‐4‐PD‐1 dual blockade hazard ratio (HR: 0.56; 95% CrI: 0.33–0.97; probability better 96.2%), whereas BRAF single agent ranked close to CTLA‐4‐PD‐1 blockade. For RR, BRAF‐MEK combination was superior to all treatments including CTLA‐4‐PD‐1 (OR: 2.78; 1.18–6.30; probability better 97.1%). No OS data were available for CTLA‐4‐PD‐1 blockade at the time of systematic review, although PFS and RR results suggested that this combination could also bring meaningful benefit. PD‐L1 expression, as presently defined, failed to inform patient selection to PD‐1‐based immunotherapy. BRAF‐MEK combination seemed an optimal therapy for BRAF‐mutated patients, whereas PD‐1 inhibitors seemed optimal for BRAF wild‐type patients. Longer follow‐up is needed to ascertain the role of CTLA‐4‐PD‐1 blockade. Immunotherapy biomarkers remain as an unmet need.

## Introduction

Recent groundbreaking discoveries in tumor biology and immune surveillance have yielded effective molecularly targeted therapies and immune agents, changing the scenario from one of poor responses and short survival to a completely new reality of high response rates, prolonged disease control, and the possibility of talking of a cure for some patients 1, 2, 3, 4, 5. Blocking the BRAF‐MEK pathway–commonly hyperactive in melanoma–has proved worthwhile. A sizeable number of trials have shown that BRAF inhibitors (BRAFi) and MEK inhibitors (MEKi) improve clinical outcomes when compared to chemotherapy 6, 7, 8, 9, 10, 11, 12. The role of the immune system in controlling melanoma is well established and immune checkpoint inhibitors have shown promise in reinvigorating the immune system, successfully showcasing the enormous potential of drugs that manipulate immune surveillance for the first time in oncology 13, 14, 15, 16, 17.

These positive results have opened new avenues in the treatment of melanoma patients and, as expected, added layers of complexity to management of patients with advanced disease. A number of studies have compared competing treatments to one another, but an overall ranking of possible interventions is lacking. The number of options has grown markedly and defining the best therapeutic plan for a particular patient is now a formidable task. This Bayesian network meta‐analysis of randomized controlled trials aims to establish relative efficacy of immunotherapy, molecularly targeted therapies, and chemotherapy, either alone or in combination, in patients with advanced or metastatic melanoma with a view to support and improve the therapeutic decision‐making process.

## Patients and Methods

### Search strategy

We searched PubMed, Embase, Clinicaltrials.gov, Cochrane Central Register of Controlled Trials, World Health Organization International Trial Registry, drugs at FDA, and Society of Melanoma Research, ASCO, ESMO, and ECCO meetings using a combination of broad terms related to melanoma and drug therapy, namely melan*, random*, immunotherapy, BRAF*, MEK*, and chemotherapy (full list of terms in appendix). References in recovered studies and relevant reviews were also screened. Databases were searched from their inception until December 21st 2015. No language restrictions were applied. We followed a predefined protocol (PROSPERO number CRD42016038618) in accordance with the PRISMA guideline for network meta‐analysis.

### Study selection

Two reviewers independently searched databases (JL, GL) and assessed eligibility of studies based on abstracts and full texts, resolving disagreements by consensus. Eligible studies were (1) randomized controlled trials enrolling patients with metastatic or advanced melanoma and describing outcomes of interest, (2) randomized patients to chemotherapy, targeted therapy against the BRAF/MEK axis or immunotherapy (not vaccine, viral therapy or biochemotherapy), and (3) BRAF and/or MEK inhibitor trial restricted inclusion to patients known to harbor BRAF mutations. Second‐line BRAF‐MEK inhibitor studies were eligible if the first‐line therapy had not been BRAF‐targeted therapy. Studies with insufficient follow‐up (≤6 months) or comparing different chemotherapy regimens were excluded. In the case of duplicated publication on the same study, the most up‐to‐date data were used. We acknowledged that inclusion criterion (4) would exclude NRAS‐mutated patients.

### Data extraction

Two authors (MG, BH) independently retrieved data from randomized controlled trial (RCT) full publications and relevant appendices, guided by an extraction form (Data S1). Disagreements were resolved by consensus.

### Outcomes of interest

Hazard ratios for overall survival (OS) and progression‐free survival (PFS), and odds ratios for response rate (RR), were collected or calculated for all included RCTs. We abstracted data from original intention‐to‐treat multivariate analysis whenever possible; thus, avoiding those derived from landmark analysis or solely based on median comparisons. We adhered to the definition of progression and the criteria used by each trial 18.

### Data synthesis and statistical analysis

Network meta‐analysis was performed in a hierarchical Bayesian random‐effects model, with relative efficacy measures, hazard and odds ratios, analyzed on the log‐scale and random effects for study. The network framework allows for synthesizing direct and indirect evidence into a single effect size. Indirect comparisons can be obtained from estimates of trials with common arms. Samples from the posterior distribution of the parameters were generated via Markov Chain Monte Carlo implemented through JAGS within R (http://mcmc-jags.sourceforge.net/). Detailed description of the Bayesian meta‐analysis model is provided in (Data S1).

We calculated posterior mean hazard and odds ratios for relative efficacy of each therapy, along with 95% credible intervals, 95% predictive intervals, and probabilities of each treatment being better (probability better) than a reference. Therapies which achieved the combined benchmarks (1) overall survival (OS) posterior mean HR ≤ 0.8 with probability better ≥ 80% as compared to chemotherapy, (2) PFS posterior mean HR ≤ 0.6 with probability better than chemotherapy ≥ 90%, and (3) response rate (RR) posterior mean OR ≥ 3.0 with probability better than chemotherapy ≥ 95% were deemed to have a meaningful benefit as compared to chemotherapy 19.

We tested the hypothesis that BRAF mutation status alters relative efficacy of immunotherapy. Interactions between BRAF mutation status and relative efficacies were incorporated in the model. We also tested the hypothesis that PD‐L1 expression affects relative efficacy of immunotherapies CTLA‐4‐PD‐1 dual blockage, PD‐1 blockage and CTLA‐4 blockage. We adhered to the trial definition of PD‐L1 positivity.

Study‐to‐study heterogeneity was summarized using predictive intervals, which provide an interval in which the relevant comparative efficacy measure would be expected to fall for a new study. Ranking and probabilities were calculated based on predicted relative effects drawn from the posterior. Quality of studies was assessed via Cochrane Collaboration's tool for assessing risk of bias in randomized trials 20. Publication bias was graphically assessed via funnel plot.

## Results

### Systematic review and qualitative analysis

A total of 1750 published or presented titles and abstracts were screened. After duplicated review and discussion, 18 trials on 10 types of therapy, comprising 7596 patients, had their data extracted. All trials were multicentric and reported in English. A sizeable number of trials used chemotherapy (dacarbazine, paclitaxel or temozolomide) as control arm. Trials assessing BRAF‐MEK dual blockade used BRAFi as control arm and restricted enrollment to patients harboring BRAF mutations. When dealing with trials comparing MEK‐chemotherapy versus chemotherapy, we restricted the data to BRAF‐mutated patients. No trial performed a head‐to‐head comparison of immunotherapy versus BRAFi. The majority of excluded randomized trials failed to use BRAFi or immunotherapy as active comparator.

Two trials have been omitted from the main analysis as they have not produced relevant data; one comparing dacarbazine to dacarbazine and ipilimumab and other comparing ipilimumab to ipilimumab and sargramostim (available upon request) 16, 21. Hence, the main analysis gathered data from 16 trials with eight therapeutic nodes and 6849 patients 6, 7, 8, 10, 13, 14, 15, 22, 23, 24, 25, 26, 27, 28, 29, 30, 31, 32, 33, 34, 35, 36.

All included evidence was intention‐to‐treat, based on standard analyses, from studies with low risk of bias, according to the Cochrane risk of bias tool (Fig. S1). No sign of publication bias was found using the funnel plot (Fig. S2). The schematic flowchart of systematic review is presented online (Fig. S3). Table 1 summarizes the trials included in the main analyses.

**Table 1:** Main features of included trials—(A) BRAFi or MEKi trials and (B) Immunotherapy trials

Study Acronym NCT	Population (line of therapy)	Treatments	*N*	OS HR (95% CI)	PFS HR (95% CI)	Response (%)
BREAK‐3^8^, ^24^ NCT01227889	Unresectable stage III or IV BRAF V600E mutated (1st or 2nd)	Dabrafenib 150 mg po bd	187	0.77 (0.52–1.13)	0.35 (0.20–0.61)	93 (50)
		DTIC^c^	63	Reference	Reference	4 (6)
BRIM‐3^6^, ^30^ NCT01006980	Previously untreated metastatic BRAF V600E mutated (1st)	Vemurafenib 960 mg po bd	337	0.70 (0.57–0.87)	0.38 (0.32–0.46)	192 (57)
		DTIC^c^	338	Reference	Reference	29 (9)
BRF113220^a^ ^7^, ^22^ NCT01072175	Metastatic, no previous BRAFi; BRAF mutated (1st, 2nd, 3rd)	Dabrafenib 150 mg po bd + trametinib 2 mg po od	54	0.79 (0.49–1.27)	0.39 (0.25–0.62)	41 (76)
		Dabrafenib 150 mg po bd + trametinib 1 mg po od	54	0.96 (0.57–1.60)	0.56 (0.37–0.87)	27 (50)
		Dabrafenib 150 mg po bd	54	Reference	Reference	29 (54)
coBRIM 26, 28 NCT01689519	Previously untreated; unresectable stage III or IV; BRAF mutated (1st)	Vemurafenib 960 mg po bd + cobimetinib 60 mg po od 3 weeks on 1 week off	247	0.70 (0.55–0.90)	0.58 (0.46–0.72)	172 (70)
		Vemurafenib 960 mg po bd + placebo	248	Reference	Reference	124 (50)
COMBI‐d^9^, ^10^ NCT01584648	Previously untreated; unresectable stage IIIC or IV; BRAF mutated (1st)	Dabrafenib 150 mg po bd + trametinib 2 mg po od	211	0.71 (0.55–0.92)	0.67 (0.53–0.84)	144 (68)
		Dabrafenib 150 mg po bd+ placebo po od	212	Reference	Reference	112 (53)
COMBI‐v^34^, ^35^ NCT01597908	Previously untreated; metastatic; BRAF mutated (1st)	Dabrafenib 150 mg po bd + trametinib 2 mg po od	352	0.66 (0.53–0.81)	0.61 (0.51–0.73)	226 (64)
		Vemurafenib 960 mg po bd	352	Reference	Reference	180 (51)
METRIC^23^, ^29^ NCT01245062	Unresectable stage III or IV BRAF mutated (no previous BRAFi, MEKi or ipilimumab) (1st or 2nd)	Trametinib 2 mg po od	214	0.54 (0.32–0.92)	0.42 (0.29–0.59)	47 (22)
		DTIC^c^ or Paclitaxel^f^	108	Reference	Reference	9 (8)
NCT00338130^25^	Previously untreated; unresectable stage III or IV (1st)^b^	Selumetinib 100 mg po bd continuously	45	1.65 (0.91–3.01)	1.24 (0.73–2.10)	5 (11)
		Temozolomide	28	Reference	Reference	3 (11)
NCT00936221^33^	Previously untreated; advanced BRAF‐mutated cutaneous or unknown primary (1st)^b^	Selumetinib 75 po bd + DTIC^c^	45	0.93 (0.57–1.53)	0.63 (0.40–0.98)	18 (40)
		Placebo po bd + DTIC^c^	46	Reference	Reference	12 (26)
(B)						
CheckMate 037 NCT01721746^13^	Progression after ipilimumab (and BRAFi if BRAF mutated) (2nd or further)	Nivolumab 3 mg/kg iv every 2 weeks	272	–	–	38 (32)
		Carbotaxol^d^ or DTIC^c^	133	Reference	Reference	5 (11)
CheckMate 066 NCT01721772^15^	Previously untreated; unresectable, stage III or IV non‐uveal, BRAF wild type (1st)	Nivolumab 3 mg/kg iv every 2 weeks + DTIC‐placebo	210	0.42 (0.25–0.73)	0.43 (0.34–0.56)	84 (40)
		DTIC^c^ + nivo‐placebo iv every 2 weeks	208	Reference	Reference	29 (14)
CheckMate 067 NCT01844505^27^	Previously untreated; unresectable stage III or IV; BRAF mutated (1st)	Nivolumab 3 mg/kg iv every 2 weeks + ipi‐placebo iv	316	–	0.57 (0.43–0.76)	138 (44)
		Nivolumab 1 mg/kg + Ipilimumab 3 mg/kg iv both every 3 weeks 4× then Nivolumab 3 mg/kg iv every 2 weeks	314	–	0.42 (0.31–0.57)	181 (58)
		Ipilimumab 3 mg/kg + nivo‐placebo iv every 3 weeks 4× then nivo‐placebo iv every 2 weeks	315	–	Reference	60 (19)
CheckMate 069 NCT01927419^14^, ^39^	Previously untreated; unresectable, stage III or IV (1st)	Nivolumab 1 mg/kg + Ipilimumab 3 mg/kg iv every 3 weeks 4× then Nivolumab 3 mg/kg iv every 2 weeks (BRAF wild type)	72	d	0.40 (0.23–0.68)	44 (61)
		Ipilimumab 3 mg/kg + Placebo iv every 3 weeks 4× then Placebo iv every 2 weeks (BRAF wild type)	37	–	Reference	4 (11)
		Nivolumab 1 mg/kg + Ipilimumab 3 mg/kg iv every 3 weeks 4× then Nivolumab 3 mg/kg iv every 2 weeks (BRAF mutated)	23	–	0.38 (0.15–1.00)	12 (52)
		Ipilimumab 3 mg/kg + Placebo iv every 3 weeks 4× then Placebo iv every 2 weeks (BRAF mutated)	10	–	Reference	1 (10)
Keynote 002 NCT01704287^32^	Progression after ipilimumab and BRAFi if BRAF mutated (2^nd^ or 3^rd^)	Pembrolizumab 2 mg/kg iv every 2 weeks	180	–	0.57 (0.45–0.73)	38 (21)
		Pembrolizumab 10 mg/kg iv every 2 weeks	181	–	0.50 (0.39–0.64)	46 (25)
		DTIC^c^ or paclitaxel^d^ or temozolomide^e^ or carbotaxol^f^	179	Reference	Reference	8 (4)
Keynote‐006 NCT01866319^36^	Unresectable stage III or IV (1st or 2nd)	Pembrolizumab 10 mg/kg iv every 2 weeks	183	0.58 (0.41–0.84)	0.55 (0.42–0.72)	62 (34)
		Pembrolizumab 10 mg/kg iv every 3 weeks	185	0.68 (0.47–0.96)	0.50 (0.38–0.66)	60 (33)
		Ipilimumab 3 mg/kg iv every 3 weeks 4x	181	Reference	Reference	22 (12)
NCT00257205^31^	Previously untreated; unresectable stage III or IV (1st)	Tremelimumab 10 mg/kg every 90 days	328	0.9 (0.75–1.07)	0.94 (0.81–1.11)	36 (11)
		Temozolomide^e^ or DTIC^c^	327	Reference	Reference	32 (10)

NCT, National Clinical Trial (NCT) number found on clinicaltrials.gov; *N*, number of enrolled patients; OS, overall survival; PFS, progression‐free survival; HR, hazard ratio; BRAFi, BRAF inhibitor; MEKi, MEK inhibitor; po, oral; od, once a day; bd, twice a day; iv, intravenously; ipi‐placebo, placebo matched to ipilimumab; nivo‐placebo, placebo matched to nivolumab.

a Included patients from randomized part (part C) of the trial.

b BRAF mutation‐positive data extracted from subgroup analysis.

Data available after systematic review and not included in the meta‐analysis.

c DTIC: Dacarbazine 1000 mg/kg iv every 3 weeks.

Paclitaxel: Paclitaxel 175 mg/m^2^ every 3 weeks.

d Carbotaxol: Paclitaxel 175 mg/m^2^ plus carboplatin AUC 5 both iv every 3 weeks.

e Temozolomide: temozolomide 200 mg/m^2^/d 5 days ON every 28 days.

f Carbotaxol: Paclitaxel 225 mg/kg plus Carboplatin AUC 6 both iv every 3 weeks.

### Quantitative analysis

The 16 trials were grouped across eight therapeutic nodes (6849 patients) according to type of therapy: chemotherapy, CTLA‐4 blockade (CTLA‐4i), PD‐1 blockade (PD‐1i), BRAF inhibitors (BRAFi), MEK inhibitors (MEKi), dual BRAF‐MEK inhibitors (BRAFi‐MEKi), chemotherapy‐MEKi, and dual CTLA‐4‐PD‐1 inhibitors (CTLA‐4i‐PD‐1i). Figure S4 describes the network design of treatments’ comparison. All standard chemotherapies (paclitaxel, temozolomide, dacarbazine) were gathered into a single therapeutic node (chemotherapy), with analogous collapse for PD‐1 drugs (nivolumab and pembrolizumab). BRAFi or MEKi results are restricted to BRAF‐mutated patients across all comparisons. Not all trials described all outcomes (Table 1).

### Efficacy

Three therapies achieved meaningful benefit compared to chemotherapy: PD‐1 blockade, BRAFi‐MEKi combination, and BRAFi. As evidenced by comparing the prediction and confidence intervals for OS, PFS, and RR, study‐to‐study heterogeneity was present, but broadly had little impact on posterior ranking of treatments.

### Overall survival

**Figure 1:**
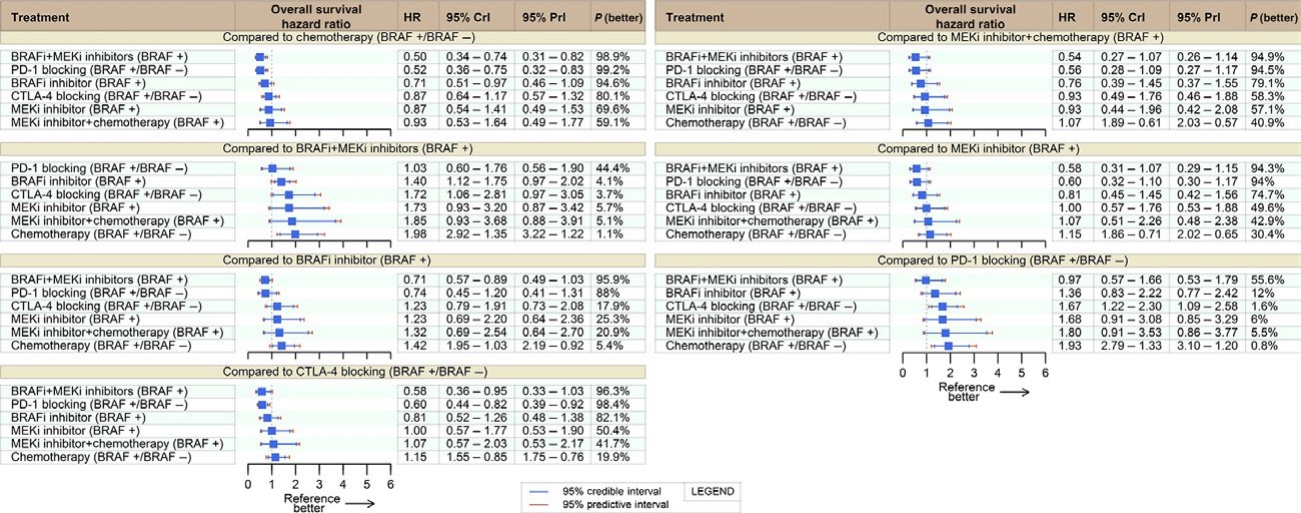
Overall survival network meta‐analysis. HR, hazard ratio; CrI, credible interval; PrI, Predictive interval; BRAF +  BRAF‐mutated patients; BRAF‐: BRAF wild‐type patients.

OS data were available for 12 (of 16) studies including 4817 patients. The results based on traditional pairwise meta‐analysis and Bayesian network meta‐analysis were aligned with no identifiable signal of inconsistency between indirect and direct approaches. Three therapies improved OS when compared to chemotherapy, BRAFi‐MEKi combination (HR: 0.50; 95% CrI: 0.34–0.74; 95% PrI: 0.31–0.82), PD‐1i (HR: 0.52; 95% CrI: 0.36–0.75; 95% PrI: 0.32–0.83), and BRAFi (HR: 0.71; 95% CrI: 0.51–0.97; 95% PrI: 0.46–1.09). PD‐1i and BRAFi‐MEKi performed similarly (HR: 1.03; 95% CrI: 0.60–1.76; 95% PrI: 0.56–1.90) with probability of BRAFi‐MEKi being superior to PD‐1i of 55.8%. Both BRAFi‐MEKi and PD‐1i had high posterior probability of outperforming all competitors. Full comparative OS results are provided in Figure 1. Given high probabilities of outperforming competitor therapies, for PFS and RR, BRAFi‐MEKi combination may be optimal for BRAF‐mutated patients, whereas PD‐1i may be optimal for BRAF wild‐type patients or selected BRAF‐mutated patients.

Despite the lack of OS data for CTLA‐4i‐PD‐1i combination at the time of systematic review, PFS and RR data were suggestive that CTLA‐4i‐PD‐1i could also achieve meaningful benefit and consequently be a top‐ranking option irrespective to BRAF status (see below) 14, 27.

### Progression‐free survival

Fifteen trials contributed to the PFS analysis. Worthy of note, the trial comparing tremelimumab (CTLA‐4i) to chemotherapy provided 6‐month time‐restricted PFS data with tumor assessments done at different time points, every 6 weeks in the dacarbazine arm and every 12 weeks in the tremelimumab arm 31. This study was not included in the PFS analysis.

Four therapies clearly stood better than chemotherapy: BRAFi‐MEKi (HR: 0.22; 95% CrI: 0.16–0.31; 95% PrI: 0.14–0.34), CTLA‐4i‐PD‐1i (HR: 0.39; 95% CrI: 0.25–0.6; 95% PrI: 0.23–0.66), BRAFi (HR: 0.39; 95% CrI: 0.29–0.52; 95% PrI: 0.26–0.59), and PD‐1i (HR: 0.5; 95% CrI: 0.4–0.64; 95% PrI: 0.34–0.73). Single agent PD‐1i and dual CTLA‐4i‐PD‐1i, both outperformed CTLA‐4i with corresponding posterior probability of 99.5% (HR: 0.53; CrI: 0.42–0.68) and 99.9% (HR: 0.42; CrI: 0.3–0.57). CTLA‐4i had similar performance to chemotherapy (HR: 0.94; CrI: 0.67–1.31).

**Figure 2:**
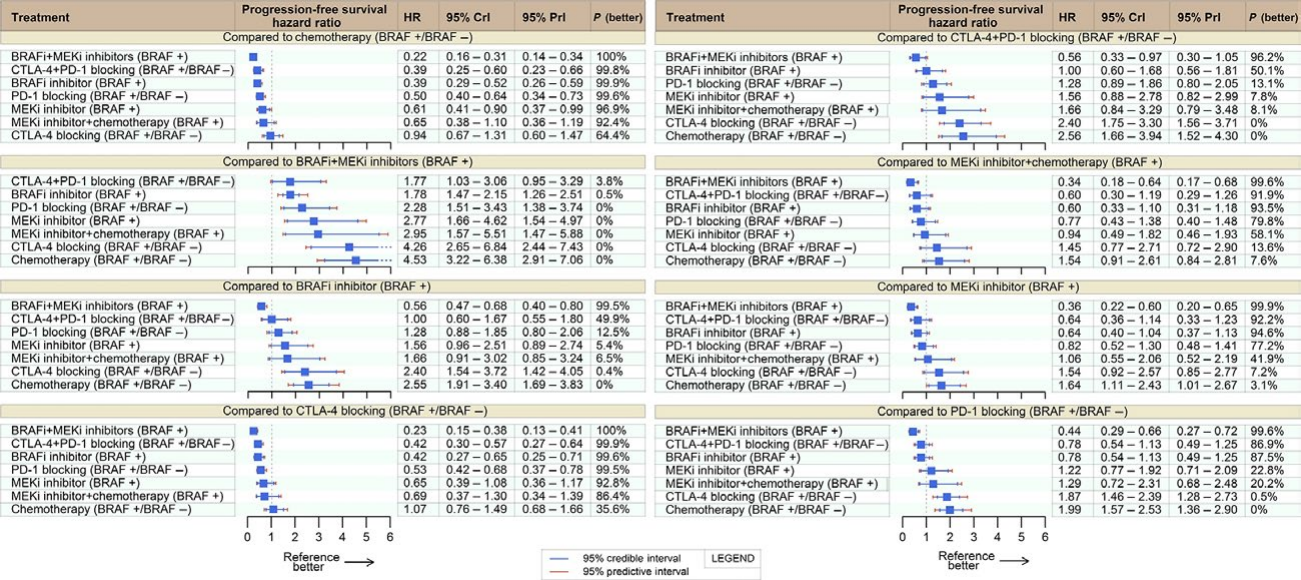
Progression‐free survival network meta‐analysis. HR, hazard ratio; CrI, credible interval; PrI, Predictive interval; BRAF+BRAF‐mutated patients; BRAF‐ BRAF wild‐type patients.

Dual BRAFi‐MEKi yielded the best PFS results with a 96.2% posterior probability of outranking the remaining options, even when compared to CTLA‐4i‐PD‐1i (HR: 0.56; CrI: 0.33–0.97). CTLA‐4i‐PD‐1i and BRAFi stood close as next options (CTLA‐4i‐PD‐1i vs. BRAFi HR: 1.00; 95% CrI: 0.6–1.67), both probably above single agent PD‐1i. Full comparative PFS results are provided in Figure 2.

### Response rate

**Figure 3:**
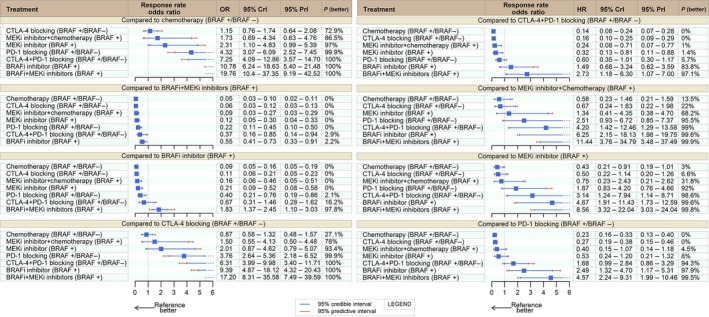
Response rate network meta‐analysis. HR, hazard ratio; CrI, credible interval; PrI, Predictive interval; BRAF+BRAF‐mutated patients; BRAF‐:BRAF wild‐type patients.

RR data were available for all studies. Bearing in mind that response under CTLA‐4i can be a late event, we included the tremelimumab versus chemotherapy trial in this analysis. Four therapies led to meaningful benefit (OR ≥ 3.0 and probability better ≥ 95% vs. chemotherapy): BRAFi‐MEKi (HR: 19.76; 95% CrI: 10.45–37.35; 95% PrI: 9.19–42.52), BRAFi (HR: 10.78; 95% CrI: 6.24–18.63; 95% PrI: 5.4–21.48), CTLA‐4i‐PD‐1i (HR: 7.25; 95% CrI: 4.09–12.86; 95% PrI: 3.57–14.7), and PD‐1i (HR: 4.32; 95% CrI: 3.07–6.09; 95% PrI: 2.52–7.45). Full comparative RR results are presented in Figure 3.

Dual BRAFi‐MEKi therapy topped best with at least 97.1% posterior probability of being superior to any other treatment: CTLA‐4i‐PD‐1i (OR: 2.73; CrI: 1.18–6.3), CTLA‐4i (OR: 17.2; CrI: 8.31–35.58), PD‐1i (OR: 4.57; CrI: 2.24–9.31), MEKi (OR: 8.56; CrI: 3.32–22.04), and BRAFi (OR: 1.83; CrI: 1.37–2.45). For BRAF‐mutated patients, the second best option was BRAFi. CTLA‐4i‐PD‐1i dual checkpoint blockade had a 94.3% posterior probability of being superior to single agent PD‐1i (OR: 1.68; 95% CrI: 0.99–2.84).

PD‐L1 expression and BRAF mutational status as biomarkers of response to immunotherapy.

The Bayesian network meta‐analysis failed to identify any relevant impact of BRAF mutation status on efficacy of immunotherapy treatments for OS, PFS, or RR in all subsets sought. The hazard ratios, 95% credible and predictive intervals of BRAF‐mutated and wild‐type patients were superimposable, which negates any role of BRAF status as a predictor of benefit of immunotherapy (Appendix S1).

Two immunotherapy trials provided information on outcomes according to PD‐L1 status 14, 27. As the definitions of positive and negative tumor PD‐L1 expression as well as the laboratory methods used to ascertain them were not homogenous across the two PD‐1 trials (Nivolumab: at least 5% of tumor cells with PD‐L1 at any intensity at the membrane; Pembrolizumab: >1% tumor cells with membranous PD‐L1 expression), we accepted the trials’ original cutoffs.

For both PFS and RR, the Bayesian network meta‐analysis failed to show any relevant impact of PD‐L1 status on efficacy of CTLA‐4i‐PD‐1i, PD‐1i, or CTLA‐4i. The hazard ratios and 95% CrIs of PD‐L1 positive and PD‐L1 negative patients overlapped, failing to identify any difference according to PD‐L1 status (Appendix S2). The posterior probability that PD‐L1 positive patients had better efficacy under CTLA‐4i‐PD‐1i, PD‐1i, or CTLA‐4i, (probability PD‐L1+ better) was from 44% to 56% for PFS, and 62% to 83% for RR.

## Discussion

This meta‐analysis synthesizes the wealth of information on immunotherapy and BRAFi/MEKi for advanced melanoma, producing a ranking of the drugs currently available. The network approach attempts to circumvent the absence of direct comparisons among the many available options, notably the comparison of immunotherapy to BRAF‐MEK inhibition and among immunotherapies. The present meta‐analysis suggests that dual BRAFi‐MEKi is the most effective in improving OS, PFS, and RR of BRAF‐mutated patients, outperforming other treatments.

Among the BRAF‐MEK axis inhibition options, single‐agent BRAFi ranked below BRAFi‐MEKi combination, but could still offer higher benefits than single MEKi. These findings may prompt inquiry into how to manage dose reduction of MEKi and BRAFi in the event of toxicities likely to be caused by both drugs. However, clinically relevant this question is, it is beyond the scope of our study to provide such practical guidance.

Appraising the PFS and RR scenarios, it was conceivable that BRAFi‐MEKi would dominate them, as BRAF‐MEK inhibition was already known to produce frequent and rapid responses, whereas immunotherapy may take longer to produce sustained tumor shrinkage and even lead to unconventional response patterns not properly captured by the standard response assessments 18, 37, 38. CTLA‐4i epitomized the immune response pattern: failed to improve PFS and RR when compared to chemotherapy, but prolonged OS, as the original trials suggested 16, 31. Our findings underscore the perception that, standard PFS assessment may not be the best way to capture anti‐tumor activity of immunotherapy. Nevertheless, it is noteworthy that dual BRAFi‐MEKi also stood as the best option with regard to OS, even when compared to single‐agent PD‐1i.

Notwithstanding the BRAF‐MEK inhibition dominance, PD‐1 blockade still ranked high in terms of OS, PFS, and RR. Hence, PD‐1i may be an attractive option for BRAF wild‐type patients and even for BRAF‐mutated patients, as it ranked in second to BRAFi‐MEKi. OS results for combined CTLA‐4‐PD‐1 immune checkpoint inhibition are not yet mature and longer follow‐up may change the order of top‐ranked therapies. Some very recent results have started to become available with promising long‐term survivorship with dual immune checkpoint blockade 39. Those findings seem to embody the preliminary reports of prolonged disease control under immunotherapy 5.

We could not confirm the role of PD‐L1 as a biomarker of response to PD‐1i‐based therapy. As currently tested, tumor PD‐L1 expression did not better inform the patient selection for PD‐1‐based therapy, both PD‐L1 positive and negative patients derived substantial benefit from PD‐1‐based therapy. This finding somewhat diverged from the realms of other tumors, showcasing the particular features of immune response within each tumor type 40. Also, our results failed to show any impact of BRAF status on response to PD‐1 therapy, confirming previous findings 41.

Several issues may be implicated on the lack of surrogacy of PD‐L1 expression. The simplest one would be statistical power constrained by a small sample size. This indeed could have played a role, however, more than 800 patients—evenly divided between PD‐L1 positive and negative—provided data for this analysis. Another possibility would be the use of inadequate cutoffs. To properly assess this, individual patient data would be required. However, even if such data were available, the different antibodies and techniques would require careful consideration. Harmonization of laboratory methods should be enacted first, as is already occurring in lung cancer with the different PD‐1/PD‐L1 agents.

Lastly, baseline PD‐L1 expression at a single tumor site may not be capable of fully capturing the complexity of anti‐PD‐1‐led orchestration of immune system dynamics. It is conceivable that resetting a whole system—in the case of immune system—might be multilayered and continuously changing.

The quest for excellent patient selection is key. Better patient selection transcends optimizing clinical outcomes. It can improve financial resource allocation, a real‐world hurdle to be crossed when new technologies are under consideration. Furthermore, identifying the most likely patients for immunotherapy will spare the nonresponders from fairly toxic therapies. The results of cooperative work on other tumors may enhance our understanding on this important topic 42, 43, 44, 45, 46.

Given the number of therapeutic options currently available for advanced melanoma, the sequencing of drugs is another crucial question. The wealth of information organized by this meta‐analysis may shed light on the long‐term therapeutic plan for melanoma patients. These nuances of clinical management are yet to be defined. However, we believe that clinicians will now be better informed for the decision‐making process. Definitive results on sequencing of the various therapeutic options will add to the knowledge base 47, 48.

A major clinical concern is the effectiveness of immunotherapy after progressing under previous BRAF‐MEK treatment. Two immunotherapy trials enrolled patients who had progressed while on BRAF‐targeted therapy 13, 32. No sign of loss of efficacy was identified with the use of PD‐1 drug among this group of patients as compared to BRAF therapy‐naive patients. Such findings must be further validated and the opposite drug order also appraised, the latter being the question of active trials 48.

This meta‐analysis faced several shortcomings inherent to the methodology applied. We had no access to individual patient data, precluding a more detailed appraisal of outcomes and patients’ characteristics. This is especially true for assessment of the role of PD‐L1 expression, volume of disease, and presence of other known prognostic markers 49, 50. We concentrated on efficacy foregoing analysis of toxicity, another major practical concern on clinical grounds. The different cutoffs used for defining PD‐L1 status hindered a more robust analysis of its relevance. The absence of overall survival data for CTLA‐4‐PD‐1 trials is a major shortcoming and hopefully more data will become available in the near future 39. Also, for the sake of simplicity, we analyzed all drugs in the same therapeutic node as identical (for instance tremelimumab and ipilimumab as CTLA‐4i prototypes). Furthermore, the duration of response could not be formally assessed as the original trials lacked enough information for a comprehensive appraisal.

Another concern was the publication and trial quality biases. We sought the most relevant databases in order to collect all published and presented trials so far, checked their references and references from relevant reviews and followed Cochrane′s guidelines on the topic. Also, we preplanned the inclusion of BRAFi or immunotherapy trials in order to concentrate on the most promising therapies; hence, some randomized trials testing other targeted therapies, such as sorafenib, oblimersen, or endothelin inhibitors were not meta‐analyzed. Trials enrolling personalized therapy to other targets, such as NRAS‐mutant tumor, were not included 51. With regard to the quality of trials included, nearly all trials were ascribed as high quality according to the Cochrane risk of bias tool, with the lack of placebo as the commonest source of likely bias.

Furthermore, it is conceivable that gathering different drugs with different doses and regimens in the same node could lead to heterogeneity, and some heterogeneity was found among the several comparisons made. Nevertheless—and most importantly—direct comparison results were in line with the network results and the impact of heterogeneity on the ranking of therapy options was minimal.

In spite of all those shortcomings listed above, we were able to formally compare different therapies and provide a clear rank of efficacy of the many available options for advanced melanoma. Abstracting all this sizeable amount of information, combined BRAFi‐MEKi‐targeted therapy seems to be a sound option at the present—even in light of emerging results of immune therapy—for BRAF‐mutant patients. Longer follow‐up in dual immune checkpoint trials coupled with further analysis of immune markers have the potential to further enhance outcomes in advanced melanoma.

## Conflict of Interest

None declared.

## Supporting information


**Data S1.** Full search strategy for PubMed.

**Figure S1.** Cochrane risk of bias tool.

**Figure S2.** Funnel plot of publication bias.

**Figure S3.** PRISMA flowchart of systematic review of studies included in the Bayesian network meta‐analysis.

**Figure S4.** Network diagram of therapeutic nodes.The area of the circle is proportional to the sample size of patients enrolled in each node; the width of connecting lines indicates the number of direct comparisons within trials. Chemo: chemotherapy; *: MEKi + chemotherapy; **: CTLA‐4i‐GMCSF; ***: CTLA‐4‐chemotherapy; Green circles: immunotherapy nodes; Orange circles: BRAFi or MEKi‐based nodes; Blue circle: chemotherapy node. Number of patients in each node: CTLA‐4i: 1172; PD‐1i: 1527; CTLA‐4i‐PD‐1i: 409; CTLA‐4‐chemotherapy: 250; CTLA‐4i‐GMCSF: 123; MEKi single agent: 259; Chemotherapy: 804; BRAFi single agent: 1390; BRAFi + MEKi: 918; MEKi + chemotherapy: 45.

**Appendix S1.** Posterior probability that BRAF‐mutated patients had better outcomes than BRAF wild type under immunotherapy.

**Appendix S2.** Posterior probability that PD‐L1‐positive patients had better outcomes on immunotherapy.

## References

[1] Eggermont, A. M. , A. Spatz , and C. Robert . 2014 Cutaneous melanoma. Lancet 383:816–827. 24054424 10.1016/S0140-6736(13)60802-8

[2] Eigentler, T. K. , U. M. Caroli , P. Radny , and C. Garbe . 2003 Palliative therapy of disseminated malignant melanoma: a systematic review of 41 randomised clinical trials. Lancet Oncol. 4:748–759. 14662431 10.1016/s1470-2045(03)01280-4

[3] Harriesa, M. , J. Malvehyb , C. Lebbec , L. Herond , J. Amelioe , Z. Szabof , et al. 2016 Treatment patterns of advanced malignant melanoma (stage III‐IV) ‐ A review of current standards in Europe. Eur. J. Cancer 60:179–189. 27118416 10.1016/j.ejca.2016.01.011

[4] Middleton, M. R. , S. Dalle , J. Claveau , et al. 2016 Real‐world treatment practice in patients with advanced melanoma in the era before ipilimumab: results from the IMAGE study. Cancer Med. 5:1436–1443. 27118102 10.1002/cam4.717 PMC4944869

[5] Schadendorf, D. , F. S. Hodi , C. Robert , et al. 2015 Pooled analysis of long‐term survival data from phase II and phase III trials of ipilimumab in unresectable or metastatic melanoma. J. Clin. Oncol. 33:1889–1894. 25667295 10.1200/JCO.2014.56.2736 PMC5089162

[6] Chapman, P. B. , A. Hauschild , C. Robert , et al. 2011 Improved survival with vemurafenib in melanoma with BRAF V600E mutation. N. Engl. J. Med. 364:2507–2516. 21639808 10.1056/NEJMoa1103782 PMC3549296

[7] Flaherty, K. T. , J. R. Infante , A. Daud , et al. 2012 Combined BRAF and MEK inhibition in melanoma with BRAF V600 mutations. N. Engl. J. Med. 367:1694–1703. 23020132 10.1056/NEJMoa1210093 PMC3549295

[8] Hauschild, A. , J. J. Grob , L. V. Demidov , et al. 2012 Dabrafenib in BRAF mutated metastatic melanoma: a multicentre, open‐label, phase 3 randomised controlled trial. Lancet 380:358–365. 22735384 10.1016/S0140-6736(12)60868-X

[9] Long, G. V. , D. Stroyakovskiy , H. Gogas , et al. 2014 Combined BRAF and MEK inhibition versus BRAF inhibition alone in melanoma. N. Engl. J. Med. 371:1877–1888. 25265492 10.1056/NEJMoa1406037

[10] Long, G. V. , D. Stroyakovskiy , H. Gogas , et al. 2015 Dabrafenib and trametinib versus dabrafenib and placebo for Val600 BRAF mutant melanoma: a multicentre, double‐blind, phase 3 randomised controlled trial. Lancet 386:444–451. 26037941 10.1016/S0140-6736(15)60898-4

[11] Schubbert, S. , K. Shannon , and G. Bollag . 2007 Hyperactive Ras in developmental disorders and cancer. Nat. Rev. Cancer 7:295–308. 17384584 10.1038/nrc2109

[12] Seton‐Rogers, S. 2014 Therapeutics: delving deeper into resistance. Nat. Rev. Cancer 14:7. 24505615 10.1038/nrc3653

[13] Weber, J. S. , S. P. D'Angelo , D. Minor , et al. 2015 Nivolumab versus chemotherapy in patients with advanced melanoma who progressed after anti‐CTLA‐4 treatment (CheckMate 037): a randomised, controlled, open‐label, phase 3 trial. Lancet Oncol. 16:375–384. 25795410 10.1016/S1470-2045(15)70076-8

[14] Postow, M. A. , J. Chesney , A. C. Pavlick , et al. 2015 Nivolumab and ipilimumab versus ipilimumab in untreated melanoma. N. Engl. J. Med. 372:2006–2017. 25891304 10.1056/NEJMoa1414428 PMC5744258

[15] Robert, C. , G. V. Long , B. Brady , et al. 2015 Nivolumab in previously untreated melanoma without BRAF mutation. N. Engl. J. Med. 372:320–330. 25399552 10.1056/NEJMoa1412082

[16] Robert, C. , L. Thomas , I. Bondarenko , et al. 2011 Ipilimumab plus dacarbazine for previously untreated metastatic melanoma. N. Engl. J. Med. 364:2517–2526. 21639810 10.1056/NEJMoa1104621

[17] Andtbacka, R. H. , H. L. Kaufman , F. Collichio , et al. 2015 TalimogeneLaherparepvec Improves Durable Response Rate in Patients With Advanced Melanoma. J. Clin. Oncol. 33:2780–2788. 26014293 10.1200/JCO.2014.58.3377

[18] Wolchok, J. D. , A. Hoos , S. O'Day , et al. 2009 Guidelines for the evaluation of immune therapy activity in solid tumors: immune‐related response criteria. Clin. Cancer Res. 15:7412–7420. 19934295 10.1158/1078-0432.CCR-09-1624

[19] Salanti, G. , A. E. Ades , and J. P. Ioannidis . 2011 Graphical methods and numerical summaries for presenting results from multiple‐treatment meta‐analysis: an overview and tutorial. J. Clin. Epidemiol. 64:163–171. 20688472 10.1016/j.jclinepi.2010.03.016

[20] Higgins, J. P. , D. G. Altman , P. C. Gotzsche , et al. 2011 The cochrane collaboration's tool for assessing risk of bias in randomised trials. BMJ 343:d5928. 22008217 10.1136/bmj.d5928 PMC3196245

[21] Hodi, F. S. , S. Lee , D. F. McDermott , et al. 2014 Ipilimumab plus sargramostim vs ipilimumab alone for treatment of metastatic melanoma: a randomized clinical trial. JAMA 312:1744–1753. 25369488 10.1001/jama.2014.13943 PMC4336189

[22] Daud, A. , J. S. Weber , J. A. Sosman , et al. 2015 Updated overall survival (OS) results for BRF113220, a phase I‐II study of dabrafenib alone versus combined dabrafenib and trametinib in patients with BRAF V600 metastatic melanoma (MM). ASCO Meeting Abstracts 33:9036.

[23] Flaherty, K. T. , C. Robert , P. Hersey , et al. 2012 Improved survival with MEK inhibition in BRAF mutated melanoma. N. Engl. J. Med. 367:107–114. 22663011 10.1056/NEJMoa1203421

[24] Hauschild, A. , J. Grobb , L. Demidov , et al. 2014 1092PD an update on overall survival (OS) and follow‐on therapies in break‐3, a phase III, randomized trial: dabrafenib (D) vs. dacarbazine (DTIC) in patients (PTS) with braf V600E mutation‐positive metastatic melanoma (MM). Ann. Oncol. 25:iv378.

[25] Kirkwood, J. M. , L. Bastholt , C. Robert , et al. 2012 Phase II, open‐label, randomized trial of the MEK1/2 inhibitor selumetinib as monotherapy versus temozolomide in patients with advanced melanoma. Clin. Cancer Res. 18:555–567. 22048237 10.1158/1078-0432.CCR-11-1491 PMC3549298

[26] Larkin, J. , P. A. Ascierto , B. Dreno , et al. 2014 Combined vemurafenib and cobimetinib in BRAF mutated melanoma. N. Engl. J. Med. 371:1867–1876. 25265494 10.1056/NEJMoa1408868

[27] Larkin, J. , V. Chiarion‐Sileni , R. Gonzalez , et al. 2015 Combined nivolumab and ipilimumab or monotherapy in untreated melanoma. N. Engl. J. Med. 373:23–34. 26027431 10.1056/NEJMoa1504030 PMC5698905

[28] Larkin, J. M. G. , Y. Yan , G. A. McArthur , et al. 2015 Update of progression‐free survival (PFS) and correlative biomarker analysis from coBRIM: phase III study of cobimetinib (cobi) plus vemurafenib (vem) in advanced BRAF mutated melanoma. ASCO Meeting Abstracts33:9006.

[29] Latimer, N. R. , H. Bell , K. R. Abrams , M. M. Amonkar , and M. Casey . 2016 Adjusting for treatment switching in the METRIC study shows further improved overall survival with trametinib compared with chemotherapy. Cancer Med. 5:806–815. 27172483 10.1002/cam4.643 PMC4864810

[30] McArthur, G. A. , P. B. Chapman , C. Robert , et al. 2014 Safety and efficacy of vemurafenib in BRAF(V600E) and BRAF(V600K) mutation‐positive melanoma (BRIM‐3): extended follow‐up of a phase 3, randomised, open‐label study. Lancet Oncol. 15:323–332. 24508103 10.1016/S1470-2045(14)70012-9 PMC4382632

[31] Ribas, A. , R. Kefford , M. A. Marshall , et al. 2013 Phase III randomized clinical trial comparing tremelimumab with standard‐of‐care chemotherapy in patients with advanced melanoma. J. Clin. Oncol. 31:616–622. 23295794 10.1200/JCO.2012.44.6112 PMC4878048

[32] Ribas, A. , I. Puzanov , R. Dummer , et al. 2015 Pembrolizumab versus investigator‐choice chemotherapy for ipilimumab‐refractory melanoma (KEYNOTE‐002): a randomised, controlled, phase 2 trial. Lancet Oncol. 16:908–918. 26115796 10.1016/S1470-2045(15)00083-2 PMC9004487

[33] Robert, C. , R. Dummer , R. Gutzmer , et al. 2013 Selumetinib plus dacarbazine versus placebo plus dacarbazine as first‐line treatment for BRAF mutant metastatic melanoma: a phase 2 double‐blind randomised study. Lancet Oncol. 14:733–740. 23735514 10.1016/S1470-2045(13)70237-7

[34] Robert, C. , B. Karaszewska , J. Schachter , et al. 2014 Improved overall survival in melanoma with combined dabrafenib and trametinib. N. Engl. J. Med. 372:30–39. 25399551 10.1056/NEJMoa1412690

[35] Robert, C. , B. Karaszewska , and J. Schachter , et al. 2015 Two year estimate of overall survival in COMBI‐v, a randomized, open‐label, phase III study comparing the combination of dabrafenib (D) and trametinib (T) with vemurafenib (Vem) as first‐line therapy in patients (pts) with unresectable or metastatic BRAF V600E/K mutation‐positive cutaneous melanoma. In: European Cancer Congress 2015, Viena, abstract 3301.

[36] Robert, C. , J. Schachter , G. V. Long , et al. 2015 Pembrolizumab versus Ipilimumab in Advanced Melanoma. N. Engl. J. Med. 372:2521–2532. 25891173 10.1056/NEJMoa1503093

[37] Simeone, E. , G. Gentilcore , D. Giannarelli , et al. 2014 Immunological and biological changes during ipilimumab treatment and their potential correlation with clinical response and survival in patients with advanced melanoma. Cancer Immunol. Immunother. 63:675–683. 24695951 10.1007/s00262-014-1545-8 PMC11028686

[38] Hodi, F. S. , W. J. Hwu , and R. Kefford , et al. 2016 Evaluation of immune‐related response criteria and RECIST v1.1 in patients with advanced melanoma treated with pembrolizumab. J. Clin. Oncol. 34:1510–1517. 26951310 10.1200/JCO.2015.64.0391 PMC5070547

[39] Postow, M. A. , J. Chesney , and A. Pavlick , et al. 2016 CT002: Initial report of overall survival rates from a randomized phase II trial evaluating the combination of nivolumab (NIVO) and ipilimumab (IPI) in patients with advanced melanoma (MEL). In: 2016 AACR annual meeting; 2016; New Orleans: AACR. p. CT002.

[40] Aguiar, P. N. , I. L. Santoro , H. Tadokoro , et al. 2016 The role of PD‐L1 expression as a predictive biomarker in advanced non‐small‐cell lung cancer: a network meta‐analysis. Immunotherapy 8:479–488. 26973128 10.2217/imt-2015-0002

[41] Larkin, J. , C. D. Lao , W. J. Urba , et al. 2015 Efficacy and safety of nivolumab in patients with BRAF V600 mutant and BRAF wild‐type advanced melanoma: a pooled analysis of 4 clinical trials. JAMA Oncol. 1:433–440. 26181250 10.1001/jamaoncol.2015.1184

[42] Ratcliffe, M. J. , A. Sharpe , A. Midha , C. Barker , P. Scorer , and J. Walker . 2016 A comparative study of PD‐L1 diagnostic assays and the classification of patients as PD‐L1 positive and PD‐L1 negative. In: AACR, editor. AACR annual meeting 2016; New Orleans: AACR; 2016. p. LBA‐094.

[43] Scheel, A. H. , M. Dietel , L. C. Heukamp , et al. 2016 Diagnostic PD‐L1 immunohistochemistry in NSCLC: results of the first German harmonization study. ASCO Meeting Abstracts 34:3028.

[44] No authors listed . 2015. A blueprint proposal for companion diagnostic comparability FDA‐AACR‐ASCO, In. Washington.

[45] Powles, T. , J. P. Eder , G. D. Fine , et al. 2014 MPDL3280A (anti‐PD‐L1) treatment leads to clinical activity in metastatic bladder cancer. Nature 515:558–562. 25428503 10.1038/nature13904

[46] Tumeh, P. C. , C. L. Harview , J. H. Yearley , et al. 2015 PD‐1 blockade induces responses by inhibiting adaptive immune resistance. Nature 515:568–571. 10.1038/nature13954 PMC4246418 25428505

[47] Ribas, A. , M. Butler , J. Lutzky , et al. 2015 Phase I study combining anti‐PD‐L1 (MEDI4736) with BRAF (dabrafenib) and/or MEK (trametinib) inhibitors in advanced melanoma. ASCO Meeting Abstracts 33:3003.

[48] Dabrafenib and Trametinib Followed by Ipilimumab and Nivolumab or Ipilimumab and Nivolumab Followed by Dabrafenib and Trametinib in Treating Patients With Stage III‐IV BRAFV600 Melanoma. 2016 Avaliable at: https://clinicaltrials.gov/ct2/show/study/NCT02224781 (Accessed 12 April 2016)

[49] Diem, S. , B. Kasenda , J. Martin‐Liberal , et al. 2015 Prognostic score for patients with advanced melanoma treated with ipilimumab. Eur. J. Cancer 51:2785–2791. 26597444 10.1016/j.ejca.2015.09.007

[50] Diem, S. , B. Kasenda , L. Spain , et al. 2015 Serum lactate dehydrogenase as an early marker for outcome in patients treated with anti‐PD‐1 therapy in metastatic melanoma. Br. J. Cancer 114:256–261. 10.1038/bjc.2015.467 PMC4742588 26794281

[51] Dummer, R. , D. Schadendorf , P. A. Ascierto , et al. 2016 Results of NEMO: A phase III trial of binimetinib (BINI) vs dacarbazine (DTIC) in NRAS‐mutant cutaneous melanoma. ASCO Meeting Abstracts 34:9500.

