# Supplementary material for: A systematic review and network meta‐analysis of immunotherapy and targeted therapy for advanced melanoma
Source: Cancer Med. 2017 May 1;6(6):1143–53. doi: 10.1002/cam4.1001 (PMC5463084; doi:10.1002/cam4.1001)
Supplement: Supplementary file 1 — Data S1. Full search strategy for PubMed. [file CAM4-6-1143-s001.docx]

Full search strategy for PubMed.

Detailed search terms and strategy for title/abstract: (melanom* or melanocyt*) AND (BRAF or BRAF* or *RAF or MEK1 or MEK2 or MEK* or MAPK or ERK1 or ERK2 or ERK* or R05185426 or RG7204 or PLX 4032 or vemurafenib or zelboraf or dabrafenib or tanfilar or GSK 2118436 or GSK2118436 or GSK-2118436 or JTP 74057 or trametinib or mekinist or JTP74057 or JTP-74057 or GSK1120212 or GSK1120212 or GSK-1120212 or cobimetinib or cotellic or GDC-0973 or XL518 or pd-1 or pd-l1 or pd-l2 or programmed cell death receptor or programmed cell death 1 receptor or programmed cell death 2 receptor or CD279 or CLTA-4 or Cytotoxic T-Lymphocyte-Associated Antigen 4 or Cytotoxic T Lymphocyte Associated Antigen 4 or Cytotoxic T-Lymphocyte Antigen 4 or Cytotoxic T Lymphocyte Antigen 4 or CD152 or CD28 OR ipilimumab or MDX-CTLA-4 or Yervoy or MDX 010 or MDX010 or MDX-010 or tremelimumab or ticilimumab or CP 675 or CP675 or CP-675 or CP-675,206 or CP-675206 or CP675206 or CP 675206 or pidilizumab or nivolumab or opdivo or bms-936558 or ono-4538 or ono4538 or mdx-1106 or pembrolizumab or lambrolizumab or keytruda or mk-3475) AND (random* or randomised or randomized or prospective)

Data extraction

Two authors (MG, BH) independently retrieved data from RCT full publications and relevant appendices guided by an extraction form. The items of interest were: trial name, first author, year of publication, number of patients, length of follow-up, methodology details (randomization, allocation concealment and blinding methods, use of intention to treat analysis), intervention details (drugs, doses, length of use), patient characteristics (median age, performance status, previous therapy, if any) and outcomes of interest (overall survival, progression-free survival, response rate). Disagreements were resolved by consensus.

Bayesian model details.

A hierarchical Bayesian network meta-analysis was performed using JAGS software within R (<http://mcmc-jags.sourceforge.net/>). All treatments were connected either directly or indirectly to each other in the network (Figure 1 at the main text).

The comparison of treatments was performed on the log hazard ratio (or odds ratio, for response rate): $log \theta_{i,j}\sim Normal\left( x_{i,j}^{'}\beta,\sigma_{i}^{2}+\tau^{2} \right)$ where $log \theta_{i,j}$ denotes the logarithm of hazard ratio *j* reported in study $i$*,* $x_{i,j}$denotes treatment contrast $j$ in study $i$*,* $\beta$denotes the vector of treatment effects relative to Chemotherapy*,* $\sigma_{i}^{2}$ denotes the within-study variance for study $i$, and $\tau^{2}$denotes the between-study variance in treatment comparisons. The distribution of all parameters was weighted by a distribution of prior beliefs. Parameters were given either non- or weakly informative priors letting the pooled data dominate the posterior distribution. Weakly informative priors were used for the mean treatment effects, placing 95% of the prior probability on hazard (odds) ratios between 1/10 (1/20) and 10 (20), so that the pooled data dominated the posterior distribution. Similarly, a weakly informative prior was used for the between-study variance which placed 95% of the prior probability on hazard (odds) to varying up to two-fold (five-fold) across studies. Estimates from three-arm studies were modeled in the context of a bivariate normal distribution with the same weakly informative prior on the between study variance along with an uninformative prior on the within-study correlation.

Ten chains were used with the first 100,000 iterations of each discarded as “burn-in”. Results are based on 500,000 iterations from each chain, thinned at a lag of 100. Posterior mean hazard and odds ratios for relative efficacy of each therapy, along with credible 95% intervals, predictive 95% intervals, and probabilities of each treatment being better than a reference were calculated. Therapies which achieved the combined benchmarks (a) overall survival (OS) posterior mean HR ≤ 0.8 with probability better ≥ 80% as compared to chemotherapy, (b) progression-free survival (PFS) posterior mean HR ≤ 0.6 with probability better than chemotherapy ≥ 90%, and (c) response rate (RR) posterior mean OR ≥ 3.0 with probability better than chemotherapy ≥ 95% were deemed to have a *meaningful benefit* as compared to chemotherapy.

The effectiveness of treatment $k$ relative to chemotherapy $\beta_{k}$ were given a weakly informative prior $\beta_{k}\mathcal{\sim N}\left( 0,\left( \frac{log(20)}{2} \right)^{2} \right).$ Priors for individual within study variances $\sigma_{i}^{2}$ were specified via inverse gamma distribution with reported value as its mean and variance proportional to $D_{i}$, the number of events for OS or PFS outcomes, $\sigma_{i}^{2} \sim Inverse Gamma\left( \frac{D_{i}}{2},\frac{D_{i}}{2}\sigma_{i}^{2} \right)$. For studies that did not report number of events (death for OS and progression or death for PFS), number of events were estimated by proxies as follows: for OS the assumption was that 50% of the randomized patients died, and for PFS 75% had PFS events by study cut-off. Between-study variances $\tau^{2}$ were assigned a weakly informative uniform distribution, $\tau\sim U\left( 0,\left( \frac{log(2)}{2} \right)^{2} \right)$, which allows hazard ratios to vary by up to two-fold across studies. Finally, within-study correlation among the two relative efficacy measures in the three arm CheckMate 067 trial was modeled as bivariate normal whose marginal distributions matching those described above and having a correlation coefficient, $\rho$. A non-informative prior distribution $\rho\sim U\left( 0,0.95 \right)$ was taken for $\rho$.
