# Supplementary material for: A systematic review and network meta‐analysis of immunotherapy and targeted therapy for advanced melanoma
Source: Cancer Med. 2017 May 1;6(6):1143–53. doi: 10.1002/cam4.1001 (PMC5463084; doi:10.1002/cam4.1001)
Supplement: Supplementary file 2 — Figure S1. Cochrane risk of bias tool. [file CAM4-6-1143-s002.pdf]

	Random sequence generation (selection bias)	Allocation concealment (selection bias)	Blinding of participants and personnel (performance bias)	Blinding of outcome assessment (detection bias)	Incomplete outcome data (attrition bias)	Selective reporting (reporting bias)	Other bias
Break-3 NCT01227889	+	+	−	+	+	+	+
BRF113220 NCT01072175 1mg	+	+	?	?	+	+	?
BRF113220NCT01072175 2mg	+	+	?	?	+	+	+
BRIM-3	+	+	−	?	+	+	+
CheckMate 037	+	+	−	?	+	+	+
CheckMate 066	+	+	+	+	+	+	+
Checkmate 067	+	+	+	+	+	+	+
Checkmate 067 ipi vs nivo	+	+	+	+	+	+	+
checkmate 067 ipi vs nivo-ipi	+	+	+	+	+	+	+
Checkmate 069	+	+	+	+	+	+	+
Checkmate 069 BRAFmut	+	+	+	+	+	+	+
Checkmate 069 BRAFwt	+	+	+	+	+	+	+
coBRIM	+	+	+	+	+	+	+
COMBI-d	+	+	+	+	+	+	+
COMBI-v NCT01597908	+	+	−	+	+	+	+
Ipi-DTIC vs DTIC Robert 2011	+	+	+	+	+	+	−
Ipi-GMCSF	+	+	−	?	+	+	+
Keynote 002 10mg	+	+	−	?	+	+	?
Keynote 002 2mg	+	+	−	?	+	+	?
Keynote 006 2wk	+	+	−	?	+	+	+
Keynote 006 3wk	+	+	−	?	+	+	+
METRIC NCT01245062	+	+	−	?	+	+	+
NCT00338130	+	+	−	?	+	+	+
NCT00936221	+	+	+	+	+	+	+
NCT01134614	+	+	−	?	+	+	+
Tremelimumab 2013	+	+	−	−	+	+	+
