# Supplementary figures and images for: A systematic review and network meta‐analysis of immunotherapy and targeted therapy for advanced melanoma
Source: Cancer Med. 2017 May 1;6(6):1143–53. doi: 10.1002/cam4.1001 (PMC5463084; doi:10.1002/cam4.1001)

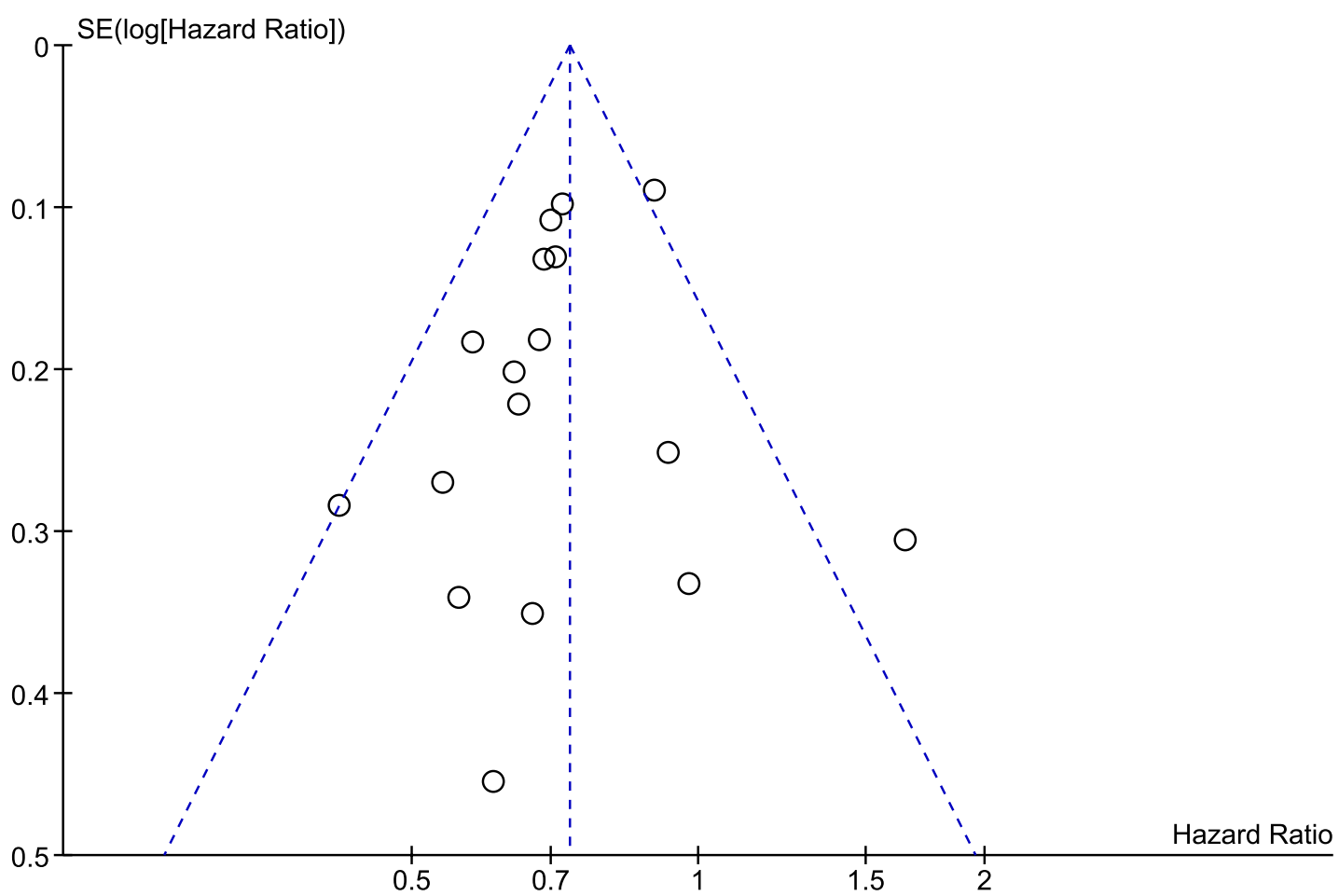

Supplement: Supplementary file 3 — Figure S2. Funnel plot of publication bias. [file CAM4-6-1143-s003.pdf]

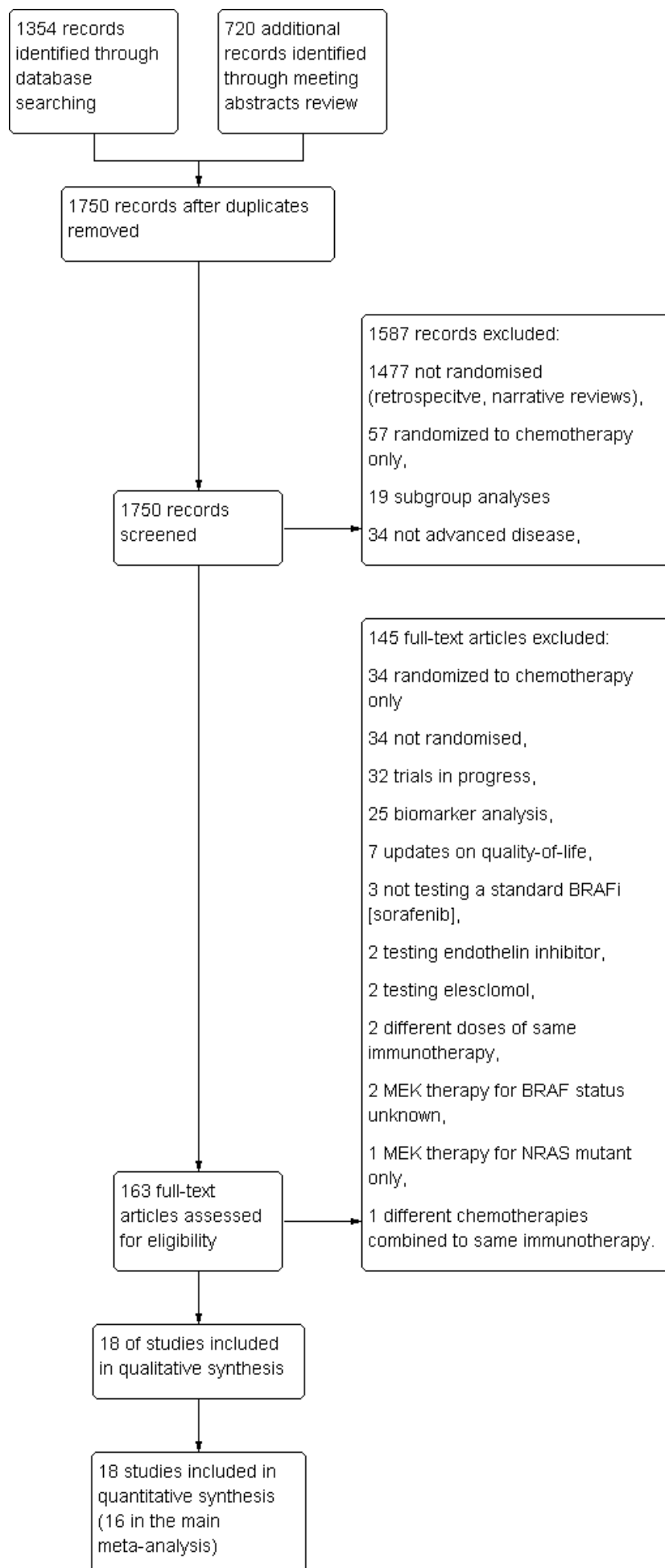

Supplement: Supplementary file 4 — Figure S3. PRISMA flowchart of systematic review of studies included in the Bayesian network meta‐analysis. [file CAM4-6-1143-s004.pdf]

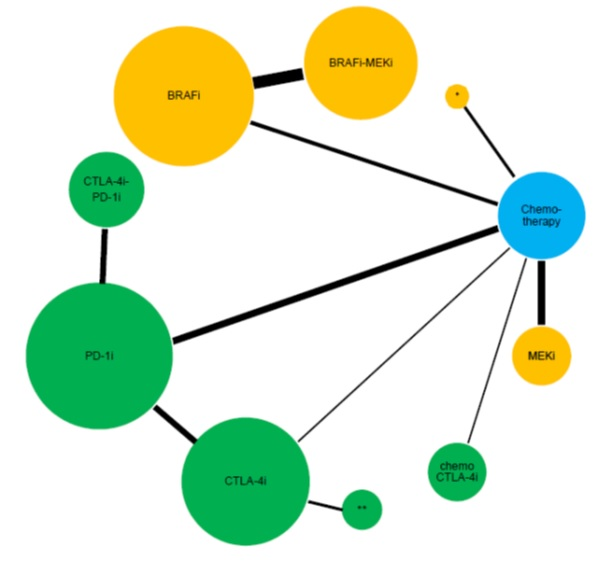

Supplement: Supplementary file 5 — Figure S4. Network diagram of therapeutic nodes.The area of the circle is proportional to the sample size of patients enrolled in each node; the width of connecting lines indicates the number of direct comparisons within trials. Chemo: chemotherapy; *: MEKi + chemotherapy; **: CTLA‐4i‐GMCSF; ***: CTLA‐4‐chemotherapy; Green circles: immunotherapy nodes; Orange circles: BRAFi or MEKi‐based nodes; Blue circle: chemotherapy node. Number of patients in each node: CTLA‐4i: 1172; PD‐1i: 1527; CTLA‐4i‐PD‐1i: 409; CTLA‐4‐chemotherapy: 250; CTLA‐4i‐GMCSF: 123; MEKi single agent: 259; Chemotherapy: 804; BRAFi single agent: 1390; BRAFi + MEKi: 918; MEKi + chemotherapy: 45. [file CAM4-6-1143-s005.tif]
