# Supplementary material for: A systematic review and network meta‐analysis of immunotherapy and targeted therapy for advanced melanoma
Source: Cancer Med. 2017 May 1;6(6):1143–53. doi: 10.1002/cam4.1001 (PMC5463084; doi:10.1002/cam4.1001)
Supplement: Supplementary file 6 — Appendix S1. Posterior probability that BRAF‐mutated patients had better outcomes than BRAF wild type under immunotherapy. [file CAM4-6-1143-s006.docx]

S9: Posterior probability that BRAF-mutated patients had better outcomes than BRAF wild type under immunotherapy.

Hazard and odds ratios are calculated as BRAF-mutated versus BRAF wild type; HR values below 1.00 indicate better outcomes for BRAF-mutated patients whereas OR values above 1.00 indicate more responses amongst BRAF-mutated patients.

| **CTLA-4i** | **Ratio** | **95% CrI** | **95% PrI** | **P(better)** |
| --- | --- | --- | --- | --- |
| OS HR | 1.01 | 0.05-20 | 0.05-20 | 49.74% |
| PFS HR | 1.69 | 0.3-10 | 0.26-10 | 27.07% |
| RR OR | 1.30 | 0.07-8.33 | 0.07-9.09 | 41% |
| **CTLA-4i-PD-1i** | **Ratio** | **95% CrI** | **95% PrI** | **P(better)** |
| OS HR | - | - | - | - |
| PFS HR | 1.64 | 0.26-10 | 0.25-11.11 | 29.65% |
| RR OR | 2.20 | 0.03-6.67 | 0.03-7.14 | 28.05% |
| **PD-1i** | **Ratio** | **95% CrI** | **95% PrI** | **P(better)** |
| OS HR | 2.08 | 0.33-12.5 | 0.32-14.29 | 21.62% |
| PFS HR | 2.08 | 0.46-9.09 | 0.44-10 | 16.94% |
| RR OR | 2.79 | 0.05-2.78 | 0.04-2.94 | 15.94% |

Abbreviations: CrI: credible interval; PrI: Predictive interval; HR: hazard ratio; OR: odds ratio; OS overall survival; PFS: progression-free survival; RR: response rate.
