# Supplementary material for: A systematic review and network meta‐analysis of immunotherapy and targeted therapy for advanced melanoma
Source: Cancer Med. 2017 May 1;6(6):1143–53. doi: 10.1002/cam4.1001 (PMC5463084; doi:10.1002/cam4.1001)
Supplement: Supplementary file 7 — Appendix S2. Posterior probability that PD‐L1‐positive patients had better outcomes on immunotherapy. [file CAM4-6-1143-s007.docx]

S10: Posterior probability that PD-L1 positive patients had better outcomes on immunotherapy.

Hazard and odds ratios are calculated as PD-L1 positive versus PD-L1 negative; HR values below 1.00 indicate better outcomes for PD-L1 positive patients whereas OR values above 1.00 indicate more response amongst PD-L1 positive patients.

| **CTLA-4i** | **Ratio** | **95% CrI** | **95% PrI** | **P(better)** |
| --- | --- | --- | --- | --- |
| **OS HR** | - | - | - | - |
| **PFS HR** | 1.19 | 0.14-10 | 0.14-10 | 43.68% |
| **RR OR** | 1.28 | 0.25-6.67 | 0.20-8.33 | 61.54% |
| **CTLA-4i+ PD-1i** | **Ratio** | **95% CrI** | **95% PrI** | **P(better)** |
| **OS HR** | - | - | - | - |
| **PFS HR** | - | - | - | - |
| **RR OR** | 1.52 | 0.25-9.09 | 0.20-11.11 | 67.66% |
| **PD-1i** | **Ratio** | **95% CrI** | **95% PrI** | **P(better)** |
| **OS HR** | - | - | - | - |
| **PFS HR** | 0.84 | 0.10-6.67 | 0.10-7.14 | 56.49% |
| **RR OR** | 2.13 | 0.45-10.0 | 0.37-12.50 | 83.31% |

Abbreviations: CrI: credible interval; PrI: Predictive interval; HR: hazard ratio; OR: odds ratio; OS: overall survival; PFS: progression-free survival; RR: response rate.
